# Imaging gray matter with concomitant null point imaging from the phase sensitive inversion recovery sequence

**DOI:** 10.1002/mrm.26061

**Published:** 2015-11-24

**Authors:** Olivier Mougin, Rasha Abdel‐Fahim, Robert Dineen, Alain Pitiot, Nikos Evangelou, Penny Gowland

**Affiliations:** ^1^ Sir Peter Mansfield Imaging Centre, School of Physics and Astronomy, University of NottinghamNottinghamNottinghamshireUnited Kingdom; ^2^ Division of Clinical NeuroscienceSchool of Medicine, University of NottinghamNG7 2RDNottinghamNottinghamshireUnited Kingdom; ^3^ Division of Radiological and Imaging SciencesUniversity of NottinghamNottinghamNottinghamshireUnited Kingdom; ^4^ School of PsychologyUniversity of NottinghamNottinghamNottinghamshireUnited Kingdom

**Keywords:** magnetic resonance imaging, multiple sclerosis, cerebral cortex, imaging, gray matter

## Abstract

**Purpose:**

To present an improved three‐dimensional (3D) interleaved phase sensitive inversion recovery (PSIR) sequence including a concomitantly acquired new contrast, null point imaging (NPI), to help detect and classify abnormalities in cortical gray matter.

**Methods:**

The 3D gradient echo PSIR images were acquired at 0.6 mm isotropic resolution on 11 multiple sclerosis (MS) patients and 9 controls subjects using a 7 Tesla (T) MRI scanner, and 2 MS patients at 3T. Cortical abnormalities were delineated on the NPI/PSIR data and later classified according to position in the cortex.

**Results:**

The NPI helped detect cortical lesions within the cortical ribbon with increased, positive contrast compared with the PSIR. It also provided improved intrinsic delineation of the ribbon, increasing confidence in classifying the lesions' locations.

**Conclusion:**

The proposed PSIR facilitates the classification of cortical lesions by providing two T_1_‐weighted 3D datasets with isotropic resolution, including the NPI showing cortical lesions with clear delineation of the gray/white matter boundary and minimal partial volume effects. Magn Reson Med 76:1512–1516, 2016. © 2015 The Authors. Magnetic Resonance in Medicine published by Wiley Periodicals, Inc. on behalf of International Society for Magnetic Resonance in Medicine. This is an open access article under the terms of the Creative Commons Attribution License, which permits use, distribution and reproduction in any medium, provided the original work is properly cited.

## INTRODUCTION

Cortical lesions, for instance in multiple sclerosis (MS), and other cortical abnormalities are difficult to distinguish with MRI, due to the need to provide three‐dimensional (3D) coverage of the whole cortex with isotropic high spatial resolution, and to provide high contrast between gray matter (GM) and white matter (WM) as well as within the cortex. This study presents a combination of the T1 weighted phase sensitive inversion recovery (PSIR) sequence 1, 2 and MP2RAGE (two inversion‐contrast magnetization‐prepared rapid gradient echo sequence) sequence, aimed at improving detection of GM abnormalities.

The PSIR sequence is an inversion recovery sequence which acquires an image in which GM and WM have equal and opposite signals [i.e., equal signals in the unsigned magnitude image, or null point image (NPI] 3, and the resulting PSIR image is reconstructed by phase correcting the NPI to give a signed dataset with double the dynamic range. PSIR 4 has been shown to improve classification of cortical lesions 1, 2 by increasing the T_1_‐weighting in the images. However, because PSIR uses a fast spin echo readout, it is difficult to acquire data at high isotropic resolution across the whole cortex in a reasonable acquisition time. MP2RAGE 5, 6 is a modification to the MPRAGE sequence used to generate images at two different inversion times, and was introduced to provide T_1_‐weighted images of the brain with increased contrast‐to‐noise ratio (CNR).

**Figure 1:**
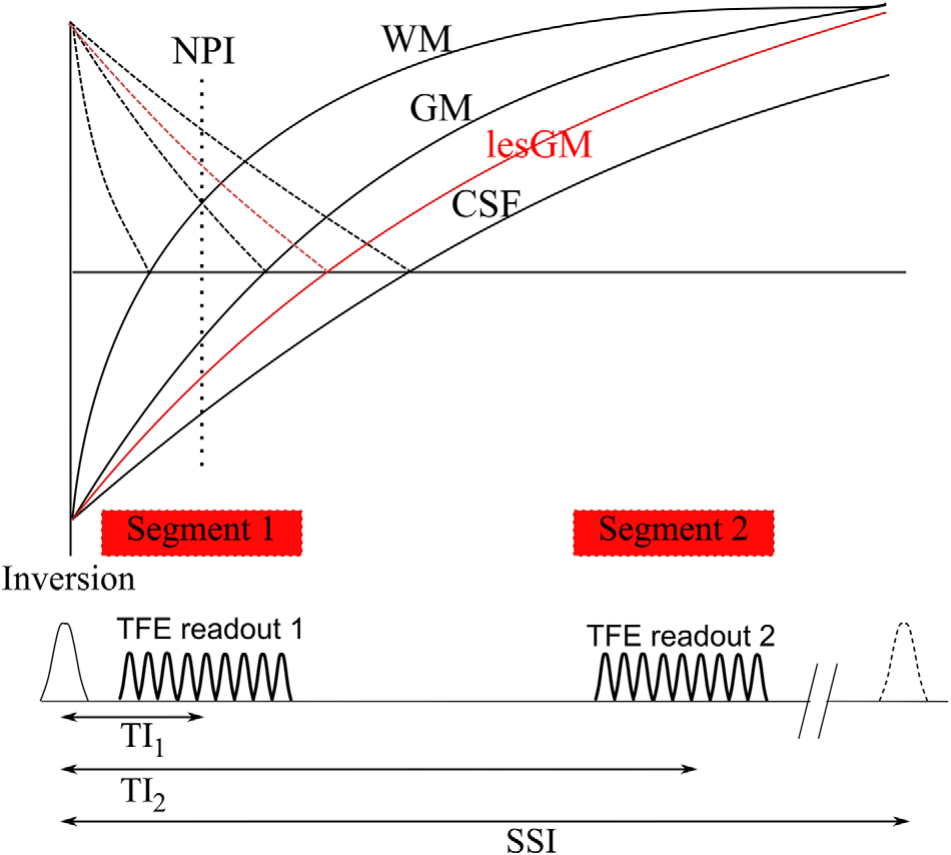
Diagram of the PSIR pulse sequence showing the evolution of the real magnetization (plain lines) and magnitude magnetization (broken lines) of the different tissues types, as well as the timing and RF pulses.

In this work, we combined these approaches by using MP2RAGE to acquire two images, the NPI and a fully recovered image (Fig. 1), to produce a PSIR with 3D isotropic coverage and simplified phase correction and bias field correction. We also adjusted the normalization used in MP2RAGE 6 to give better contrast throughout the cortical ribbon. Most importantly we retained the NPI itself, because it provides different GM/WM and intracortical contrast compared with the PSIR image and also gives a dark line at the interface between the GM and WM on the magnitude reconstructed image due to the partial volume effects.

In summary, we describe a series of simple but significant modifications to PSIR to increase its value in detecting and classifying cortical abnormalities, including making use of the NPI.

## METHODS

Eleven patients (8 females; mean age, 48 ± 9 years) with MS were recruited to a study of imaging GM lesions, and their data were used to demonstrate the efficacy of the PSIR with concomitant NPI sequence. Two patients had clinically isolated syndrome (CIS), five had relapsing remitting (RR) MS, one had primary progressive (PP) MS, and three had secondary progressive (SP) MS. Nine healthy controls (six females, mean age: 36 ± 10 years) were also recruited. The project had approval from a National Health Service Research Ethics Committee, and all subjects gave informed consent.

MRI scans were acquired using a 7 Tesla (T) Achieva Philips scanner, with a head only quadrature transmit radiofrequency (RF) coil and a NOVA 32‐channel receive coil. The PSIR sequence acquired two turbo field echo (TFE) readouts after each inversion pulse as shown in Figure 1, at TI_1_ = 780 ms (signal *SI_1_*) and TI_2_ = 2380 ms (*SI_2_*). A tailored adiabatic inversion pulse 7 was used to reduce effect of the $B_{1}^{+}$ inhomogeneities. TI_1_ was chosen to make the signals from GM and WM approximately equal and opposite, nulling voxels containing equal fractions of GM and WM (the NPI). TI_2_ was chosen to be short to reduce acquisition time but had to be long enough for the signals from WM, GM, and cerebrospinal fluid (CSF) to have recovered past the null point, as required for the reconstruction. Other parameters were shot to shot interval (SSi) 5000 ms, AP readout direction, phase encoding radial in RL and FH directions with the acquisition of 110 fast field echoes (FFE) per segment with the k‐space center being acquired in the middle of the segment, sensitivity encoding (SENSE) factors 2.2 × 1 × 2, FFE flip angle of 8° (with RF spoiling), echo time/repetition time (TE/TR) = 6/13 ms, isotropic voxel length 0.6 mm, field of view 200 × 180 × 140 mm 3, acquisition time 11.92 min. Two MS patients were also scanned using a 3T Achieva Philips scanner, with the same NPI/PSIR protocol, but with different resolution (0.8 mm isotropic) and inversion time chosen to provide a NPI at 3T (TI_1_ = 482 ms) with a total acquisition time of 9.8 min.

**Figure 2:**
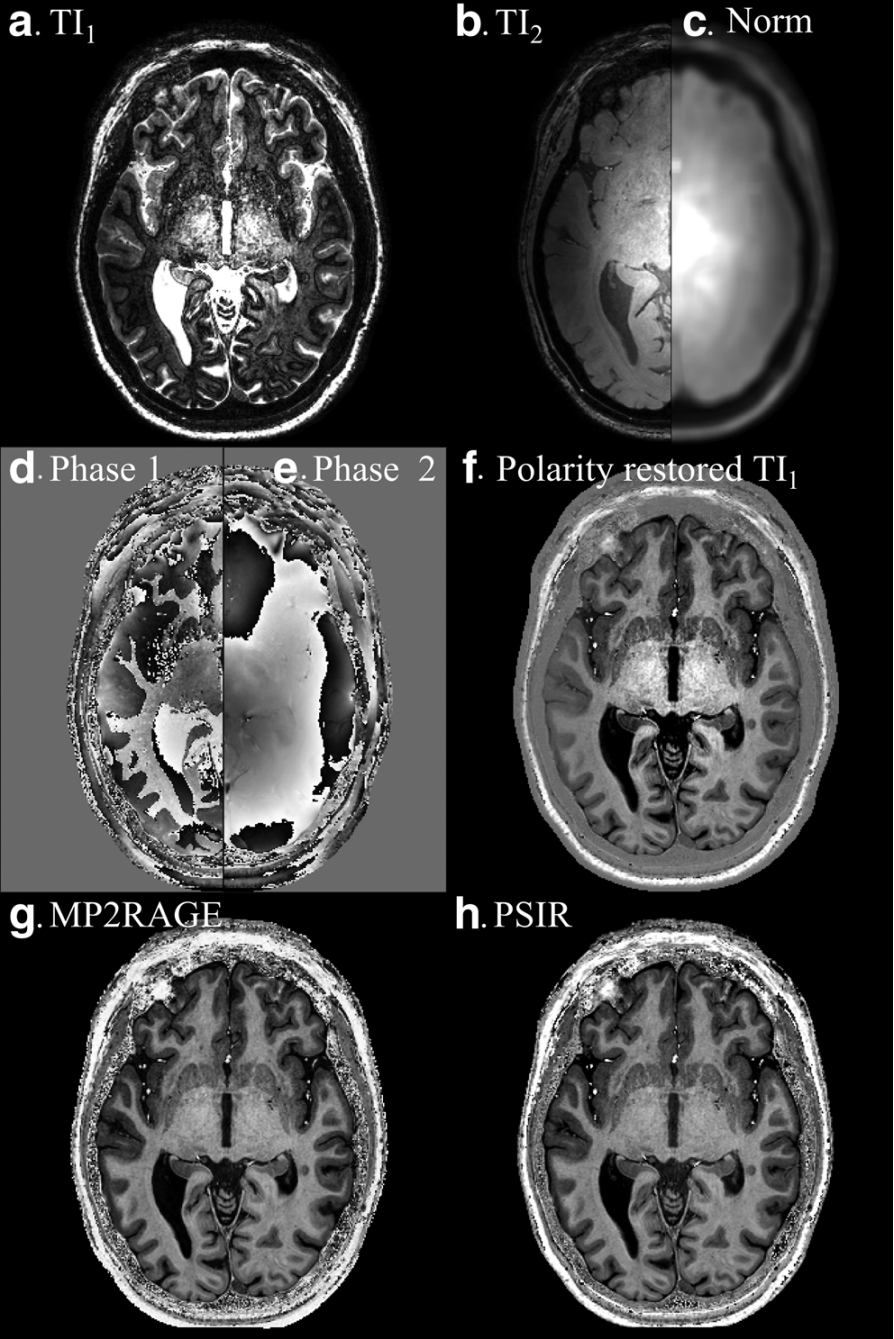
NPI image acquired at TI_1_ and TI_2_ (**a,b**) with corresponding phase maps (**d,e**) together with the corresponding smoothed data used for normalization (**c**) obtained after adding a and b together and low‐pass filtering the result. The phase corrected, unnormalized NPI image is shown (**f**) together with processed MP2RAGE image (**g**) and PSIR image (**h**), equally windowed.

Figures 2a and 2b show the images acquired at TI_1_ (NPI) and TI_2_, and Figures 2d and 2e show the corresponding phase maps. The difference in phase between Figures 2d and 2e was used to restore the modulus signal polarity for the NPI (Fig. 2f) 8. The polarity was defined as being negative if the phase change between *SI_1_* and *SI_2_* lay in the range ^π^/_2_ and ^3π^/_2_. However, alternative polarity correction method could be used. The image acquired at TI_2_ (Fig. 2b) is dominated by the effects of transmit and receive field inhomogeneities on the TFE readout, although some T_1_ weighting occurs in CSF. Therefore, the modulus images acquired at TI_1_ and TI_2_ were summed (to overcome the low signal in the CSF of the *SI*_2_), and then smoothed with a Gaussian convolution kernel (9 × 9 × 9 voxels) to reduce noise (Fig. 2c). A bias field corrected PSIR image (Fig. 2h) was produced by dividing the polarity restored NPI (Fig. 2f) by the smoothed image (Fig. 2c). This PSIR reconstruction can be summarized as:

(1) $$PSIR=\frac{\pm \left|SI_{1}\right|}{\left|SI_{1}\right|+\left|SI_{2}\right|}$$

where ± indicates polarity restoration, neglecting smoothing of the denominator. Data were also reconstructed using the MP2RAGE approach 6.

(2) $$MP2RAGE=\frac{SI_{1}^{*}\cdot SI_{2}^{}}{SI_{1}^{2}+SI_{2}^{2}}$$

where *SI_1_* ^*^ indicates the complex conjugate of *SI_1_*.

**Figure 3:**
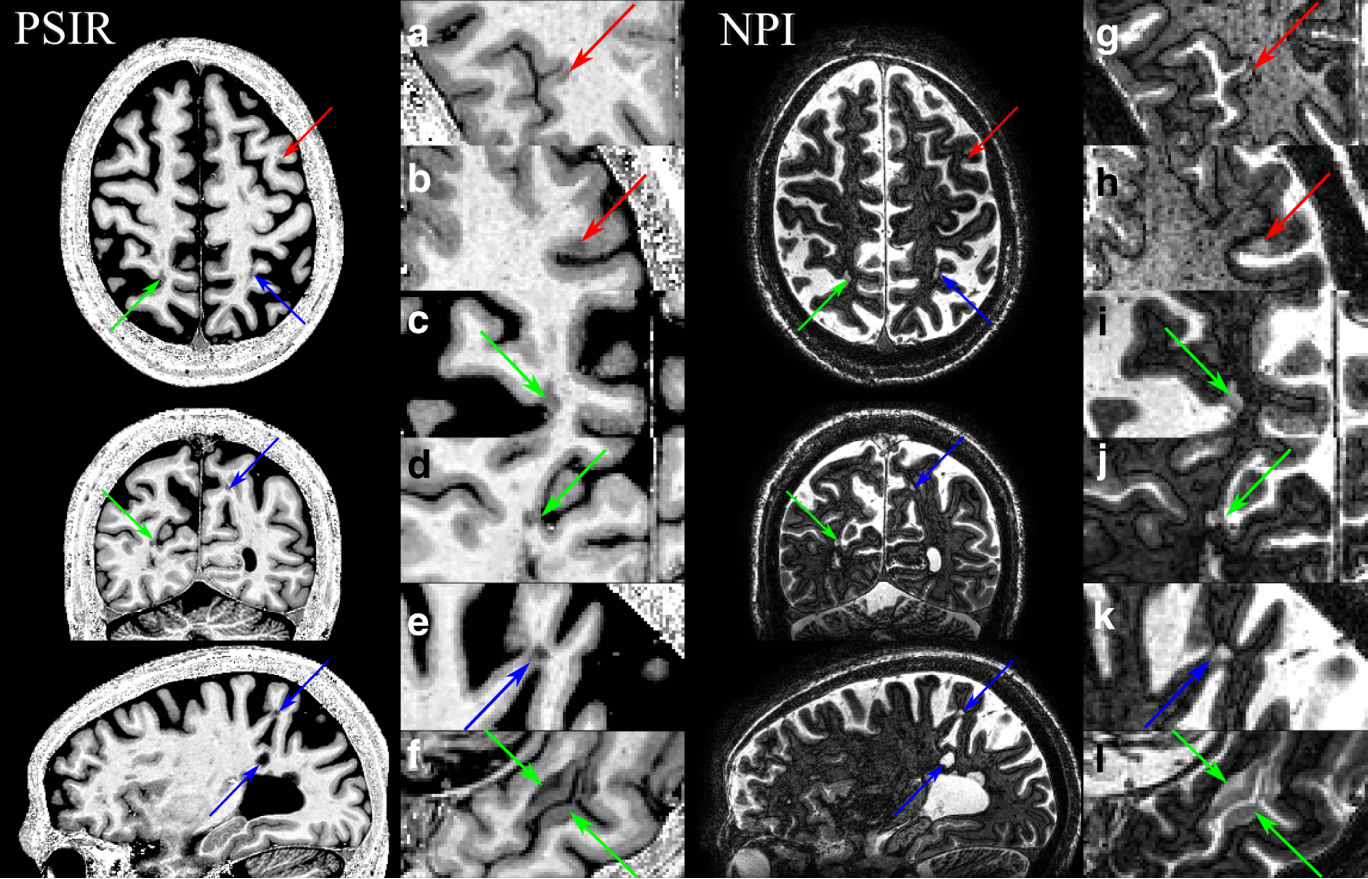
PSIR (**a–f**) and corresponding raw NPI images (**g–l**) showing cortical lesions that are hypointense/hyperintense on PSIR/NPI (red arrows) as well as mixed (green arrows) and juxtacortical lesions (blue arrows). Note the black line between the WM and GM in the NPI images, corresponding to the WM/GM boundary.

PSIR and NPI images were reviewed together by an experienced nonclinical operator (OM) to detect any abnormality involving the cortical ribbon, as presented in Figure 3. For a subset of 43 intracortical lesions randomly selected on the 11 patients, regions of interest (ROI) were manually drawn in intracortical GM (icGM) lesions as well as in Normally Appearing GM (NAGM) and WM (NAWM) nearby to the icGM lesions (less than 15 pixels from the lesion border in several slices, average volume of 177 and 370 pixels, respectively), so that CNR could be calculated for WM/GM, WM/icGM, and icGM/GM:

(3) $$CNR=\frac{\left(S_{A}-S_{B}\right)}{\sqrt{\frac{\sigma_{A}^{2}+\sigma_{B}^{2}}{2}}}$$

where S_A_ and S_B_ are the signals, and σ_A_ and σ_B_ are the standard deviations of the signals, in each ROI. CNR was compared between NPI, PSIR, and MP2RAGE images at the same location within the brain, with a balanced two‐way analysis of variance (ANOVA) test, corrected for multiple comparisons by Bonferroni correction, with a *P*‐value threshold of *P* = 0.05. The general behavior of the two normalization methods was compared by propagating errors through Eqs. [1] and [2], to calculate the signal‐to‐noise ratio (SNR) for a range of signals (−1 < *SI_1_* < 1 and (*SI_1_* < *SI_2_* < 1) and the CNR between two tissues (−1 < *SI_1_* < 1 and *SI_2_* = 1 for both tissues separately).

## RESULTS

There was no significant difference in the age or gender of MS patients and control subjects (pooled two‐sample t‐test; *P* = 0.83). Figure 3 is a typical 7T dataset demonstrating the whole brain coverage available: it illustrates how the combined use of the simultaneously acquired PSIR and NPI images helps to detect and classify intracortical lesions due to their positive contrast within the cortical ribbon on the NPI images. At the first inversion time (TI_1_) the longitudinal magnetization of cortical lesions is more negative than the GM and thus they have negative contrast on polarity restored PSIR images but positive contrast on the magnitude NPI images (Figure 3, red arrows). The dark boundary at the GM/WM interface on the NPI makes it easier to distinguish cortical from juxtacortical lesions (Figs. 3g,k). Mixed GM/WM (Figure 3 green arrows) and juxta cortical WM lesions (Figure 3, blue arrows) were more easily detected on PSIR than NPI due to the dark (bright) appearance of the lesions within WM, and GM involvement in mixed GM/WM lesions was particularly easy to detect on NPI images (Figs. 3i,j,l). The sequence could also be used at 3T to detect intracortical and juxtacortical lesions (Supporting Figure S1, which is available online).

**Figure 4:**
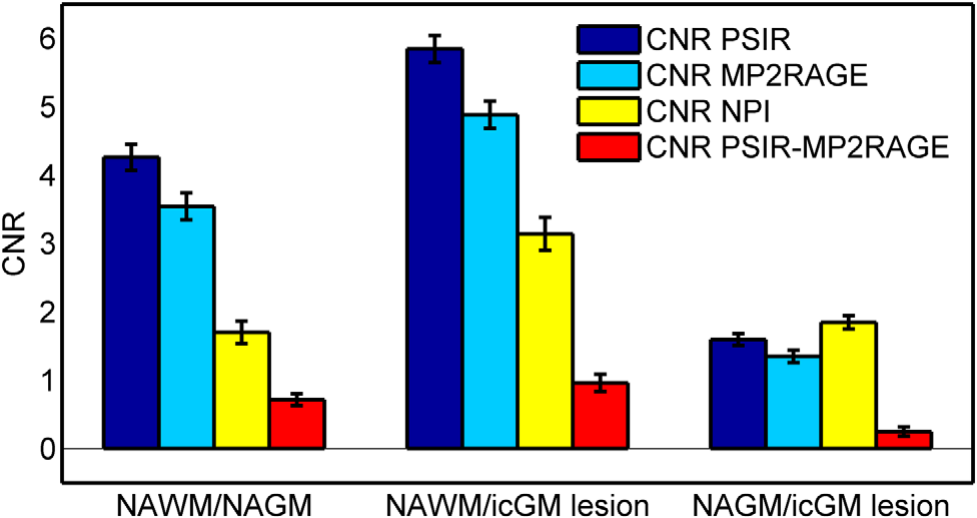
Experimental CNR obtained in 44 icGM lesions as well as adjacent NAWM and NAGM ROIs from 11 MS patients.

**Figure 5:**
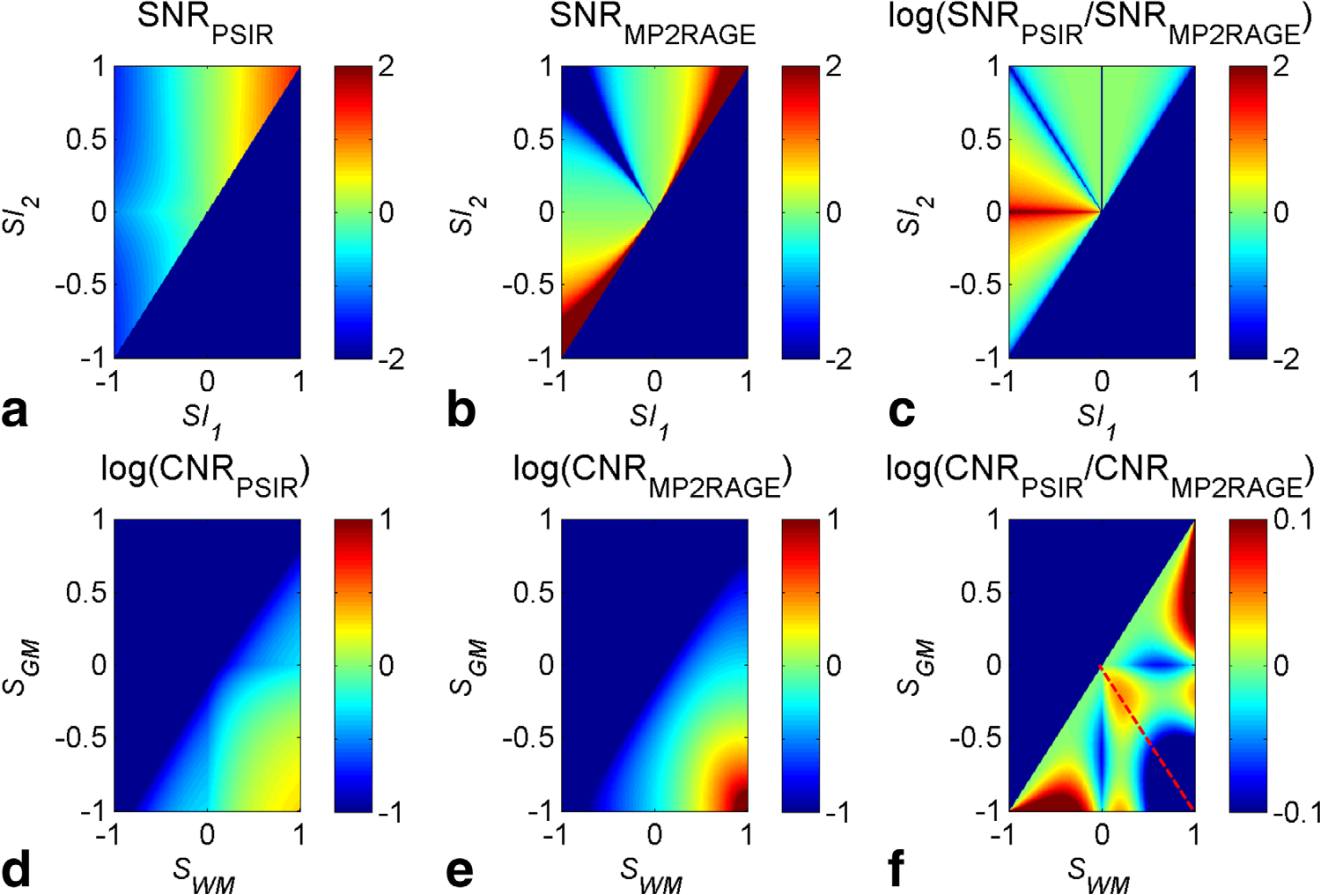
Plots showing how SNR (**a,b**) and CNR (**d,e**) vary with signals in the first (*SI_1_*) and second (*SI_2_*) image of the acquisition for the PSIR (Eq. [2]) and MP2RAGE (Eq. [3]) methods of normalization (assuming *SI_2_* = 1 for the CNR). **c,f**: Show the log of the ratio in SNR and CNR of the two reconstruction methods, with values greater than 0 indicating regions where the PSIR is superior to MP2RAGE reconstruction and the red dotted line showing S_GM_ = ‐S_WM_. The SNR and CNR plotted are relative values to be scaled by the factional SNR in the raw images.

With PSIR/NPI at 7T, 150 cortical lesions were found in 11 patients, of which 56 were classified as intracortical; 12 lesions were found in 9 healthy controls. Figure 4 shows that CNR between NAWM and NAGM was greater in PSIR than NPI (*P* < 0.0005, balanced two‐way ANOVA test, adjusted for multiple comparison by Bonferroni correction) as expected because the NPI image is designed to match the absolute signal intensity in NAWM and NAGM. The CNR between NAWM and icGM lesions was also greater in PSIR (*P* < 0.0005) than NPI. However, the CNR measured between NAGM and intracortical GM lesions was greater in NPI than PSIR (*P* < 0.001). Figure 4 also shows that CNR was greater for PSIR reconstruction compared with MP2RAGE reconstruction for all comparisons (*P* < 0.0005 for NAGM versus intracortical GM lesions), in agreement with simulation results shown in Figures 5a–f.

Figures 5a and 5b show theoretical SNR for PSIR and standard MP2RAGE normalizations (by propagating errors through Eqs. [1] and [2]); negative SNR indicates regions where the sign of the signal will be negative. Note that only half of the signal is presented as *SI_2_* cannot be less than *SI_1_*. Figure 5c shows the log of the ratio of these two plots, so that negative values indicate regions in which the SNR is greater for the MP2RAGE normalization. There is very little difference in the raw SNR if S1∼0 and S2∼1 as required to create the NPI. Figures 5d and 5e illustrates the behavior of the sequence showing how the CNR between the tissues would vary for different values of *SI_1_* assuming *SI_2_* = 1, i.e., large SSi so that all magnetization recovers before acquisition of TI_2_), and again Figure 5f shows the log of the ratio of these two plots. If the signals from each tissue are approximately equal and opposite (red line in Figure 5f), and relatively small (S_WM_∼‐S_GM_≪1), as required to produce the NPI, then the CNR is greater for PSIR. Simulation with exact relaxation times gave ∼10% greater CNR for the PSIR compared with MP2RAGE, compared with ∼20% improvement measured experimentally (Fig. 4). CNR will be greater in MP2RAGE when either tissue of interest has signal close to 0, such as at the WM/GM border, but it does not provide the concomitant raw NPI which would otherwise provide excellent contrast in this region.

## DISCUSSION

This study presented a series of modifications to the PSIR sequence to simplify the detection and classification of cortical abnormalities particularly in MS.

We modified the PSIR sequence 1, 2, which originally had a fast spin echo readout, to use a 3D fast field echo (FFE) readout as in MPRAGE 9 and MP2RAGE 6. This provides isotropic whole brain coverage, which increases sensitivity to small lesions by reducing partial volume effects, and eases detection by allowing images to be viewed in multiple orientations. The TFE readout also reduces sensitivity to B_1_ inhomogeneities and SAR at high field.

Second, PSIR usually involves acquiring an image at an inversion time between the null points of GM and WM 4, and using phase correction to recover the sign of the magnetization 8. Here, we adopted the MP2RAGE approach, interleaving this null point image with a second, fully recovered image (Fig. 2). The phase of this second image was used to restore the signal polarity in the first image 3, 8, and its magnitude was used to correct the bias field arising from both transmit and receive inhomogeneities 5.

Third, Marques et al 6 showed that using the normalization in Eq. [2] increases SNR compared with simple division of *SI_1_* by *SI_2_*, but we found that for the combination of inversion times required to form the NPI, normalizing by the sum of the magnitude signals (Eq. [1]) improved CNR for the tissues of interest, as confirmed by both simulations and experimental results. Figure 5 illustrates that the normalization methods produced different sensitivities to noise for particular combinations of signals because of the different values of the numerators in the equations (1) and (2). However, the PSIR normalization increased the CNR measured between GM and WM compared with MP2RAGE, whereas it decreased CNR in regions where the signal of interest was close to the null point, but in those regions additional information is available from the NPI. The comparison was done in the same region of interest between the different reconstructions of the same data; therefore, biological variations will be constant within subjects. Biological variation within the ROI was kept small by using small ROIs, but regional variation is expected within the brain. Nonetheless, Figures 2g and 2h show little visible difference in contrast between PSIR and MP2RAGE. Future work should focus on estimating how the two reconstructions performed compared with artifacts such as motion or large field inhomogeneities. The acquisition parameters were optimized for the proposed reconstruction using the software used in 6 (Marques, personal communication). It showed that using the TI_1_ necessary to produce the NPI, and TI_2_ necessary to allow all signals to recover past the null point in the second image, then the other sequence parameters are near optimal for both the MP2RAGE and the PSIR reconstruction (TI_2_ = 2.25 s for PSIR and TI_2_ = 2.2 s for MP2RAGE). Figure 5 provides a method of tailoring the normalization to the problem being addressed: Eq. [1] is proposed for the problem of detecting GM abnormalities. The CNR could be greater in the NPI than the processed PSIR or MP2RAGE images (Fig. 4), because the process of combining the images (in particular taking a ratio) can add noise.

Finally and most significantly, we retained the NPI acquired at TI_1_ as part of the PSIR acquisition. This is usually only used to calculate the PSIR, but it contains useful information in its own right, which can be used in combination with PSIR to help determine whether a lesion is intracortical, mixed, or juxtacortical. The T_1_ differences between the different tissues are quite large, especially at high field, which makes this NPI technique very robust to B1 inhomogeneities, as well as T_1_ variations. The visual and motor cortices known to have shorter T_1_s are still delineated by the dark line, as shown on Figure 2a and Supporting Figure S1, respectively. Simulations have shown that only a small percentage of partial voluming is necessary to create a dark boundary on the NPI. Intracortical lesions have increased CNR on the NPI compared with the PSIR image, and have positive contrast on the NPI whereas they have negative contrast on PSIR (or standard MPRAGE). Furthermore, the NPI delineates the cortical ribbon because it shows a dark line at the GM/WM boundary (where voxels contain a mixture of inverted and uninverted magnetization) 1, 3. The NPI contrast and WM/GM delineation is transferable to lower field strength, as shown in Supporting Figures S1b and S1d, albeit with lower SNR or lower resolution.

In conclusion, the 3D TFE PSIR image with its concomitant NPI provides two complementary images for use in detecting cortical lesions by providing isotropic high resolution images (0.6 mm^3^ at 7T), in three orthogonal planes, with clear delineation of the GM/WM boundary and with cortical lesions giving positive contrast in the NPI.

## Supporting information


**Supporting Figure S1.** PSIR images (**a,c**) and corresponding NPI images (**b,d**) acquired on a MS patient at 3T, showing both juxtacortical lesions (blue arrows) and intracortical lesion (red arrows).
