# Supplementary material for: Imaging gray matter with concomitant null point imaging from the phase sensitive inversion recovery sequence
Source: Magn Reson Med. 2015 Nov 24;76(5):1512–6. doi: 10.1002/mrm.26061 (PMC5082579; doi:10.1002/mrm.26061)
Supplement: Supplementary file 1 — Supporting Figure S1. PSIR images (a,c) and corresponding NPI images (b,d) acquired on a MS patient at 3T, showing both juxtacortical lesions (blue arrows) and intracortical lesion (red arrows). [file MRM-76-1512-s001.docx]

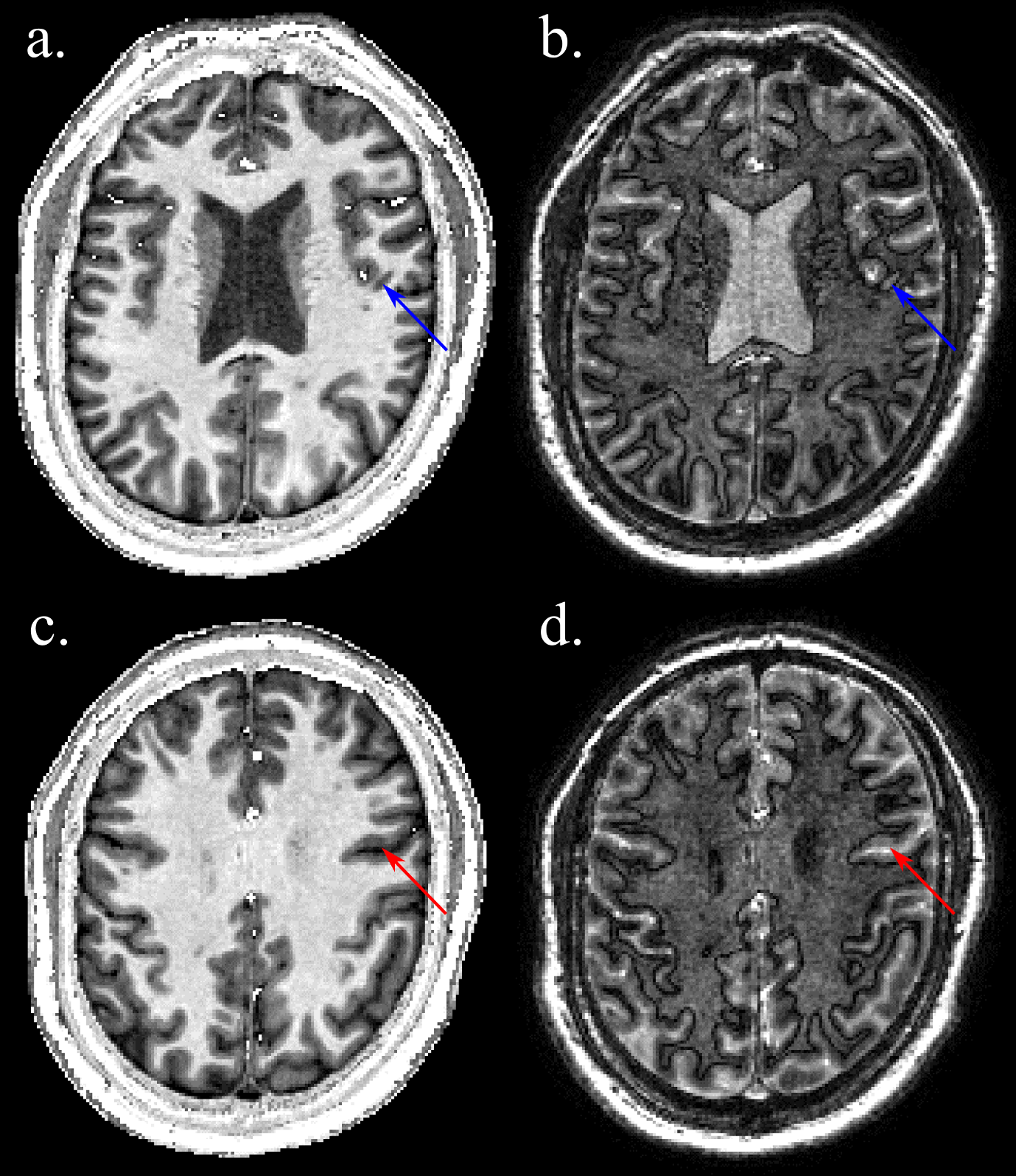


**Supporting Figure S1:** PSIR images (a and c) and corresponding NPI images (b and d) acquired on a MS patient at 3T, showing both juxtacortical lesions (blue arrows) and intra-cortical lesion (red arrows).
